# Umbilical Cord Mesenchymal Stem Cells Ameliorate Kidney Injury in MRL/Ipr Mice Through the TGF-β1 Pathway

**DOI:** 10.3389/fcell.2022.876054

**Published:** 2022-04-05

**Authors:** Chunkai Huang, Mingyao Meng, Shuo Li, Shiyuan Liu, Lin Li, Yanjun Su, Hui Gao, Shan He, Yiyi Zhao, Min Zhang, Zongliu Hou, Wenju Wang, Xiaodan Wang

**Affiliations:** ^1^ Scientific Research Department, Yan’an Hospital Affiliated to Kunming Medical University, Kunming, China; ^2^ Key Laboratory of Tumor Immunological Prevention and Treatment of Yunnan Province, Kunming, China; ^3^ Yunnan Cell Biology and Clinical Translation Research Center, Kunming, China; ^4^ Thyroid Surgery, The First Affiliated Hospital of Kunming Medical University, Kunming, China

**Keywords:** lupus nephritis, umbilical cord mesenchymal stem cells, podocytes, TGF-β1, p-Smad3, TRAF6

## Abstract

The therapeutic effects and mechanism of umbilical cord mesenchymal stem cells (UC-MSC) on kidney injury in MRL/Ipr mice were studied. UC-MSC, methylprednisolone (MP), and their combination were used to treat MRL/Ipr mice. The therapeutic effects were evaluated by renal function assessment, and HE, PAS, and Masson staining were carried out on renal tissues and visualized by electron microscopy. Subsequently, podocyte injury was detected by the presence of podocin in renal tissues by immunofluorescence. To further explore the mechanism, serum TGF-β1 was measured, and TGF-β1, p-Smad3, and TRAF6 in the renal tissue were detected by Western blotting. *In vitro*, TGF-β1 was used to stimulate podocytes, and the podocyte activity and changes in synaptopodin were observed after UC-MSC treatment. Significant improvements in renal function and pathological injury were observed in the UC-MSC group compared to the lupus nephritis (LN) model group. UC-MSC and MP treatment improved podocyte injury in MRL/Ipr mice. Western blot examination showed a significant increase in TGF-β1, p-Smad3, and TRAF6 expression in renal tissues of the LN model group, while significant downregulation of those proteins was observed in the UC-MSC group. After TGF-β1 stimulation *in vitro*, podocyte activity decreased, and UC-MSC treatment improved podocyte activity and restored synaptopodin expression. UC-MSC therapy could improve the deterioration of renal function and the pathological changes of the renal tissues in MRL/Ipr mice. Our study suggested that UC-MSC may improve kidney injury and podocyte injury in LN mice by inhibiting the TGF-β1 pathway.

## 1 Introduction

Systemic lupus erythematosus (SLE) is a multisystem disease characterized by the production of autoantibodies. Lupus nephritis (LN) is one of the most common manifestations, affecting about 40% of the patients with SLE. It is a major risk factor for morbidity and mortality, and 10% of patients with LN will develop end-stage renal disease (ESRD). Corticosteroids combined with immunosuppressors are the standard treatment regimens for LN (Gasparotto et al., 2020; Morales et al., 2021). However, some patients still have poor prognosis who are strictly treated by the aforementioned treatment regimens. Furthermore, corticosteroids and immunosuppressants may cause serious and even fatal infections, and the management of these complications is still challenging. Based on the immunomodulatory effects and cytokines secreted by mesenchymal stem cells (MSC), some clinical studies have suggested that MSC have therapeutic effects on LN (Liang et al., 2010; Gu et al., 2014; Wang et al., 2014). However, the underlying molecular mechanisms of these effects are still unclear.

Transforming growth factor-β (TGF-β) is an important mediator in renal fibrosis (Isaka, 2018; Ma and Meng, 2019). The MRL/Ipr mice showed progressive glomerulosclerosis and increased expression of TGF-β (Kong et al., 2019; Qi et al., 2020). TGF-β has three isoforms, of which TGF-β1 has been identified as the most potent mediator and convergent pathway in renal fibrosis (Hu et al., 2018). TGF-β1 was reported to be diffusely expressed in the kidney tissues of LN patients, urinary TGF-β1 was increased and strongly associated with the SLEDAI score (Badawy et al., 2020; Khalili et al., 2020; Vanarsa et al., 2020). The downstream targets of TGF-β1 mainly include the Smad and Smad-independent pathways. The equilibrium of Smad signaling is essential for TGF-β-mediated renal fibrosis, and Smad3 is pathogenic factor, while Smad2 and Smad7 play a protective role. The activation of TGF-β1/Smad signaling leads to extracellular matrix deposition and local myofibroblast generation and activation (Tang et al., 2018). In the process of TGF-β1-stimulated Smad-independent pathways, tumor necrosis factor receptor-associated factor 6 (TRAF6) plays an important role in cytokine production and the inflammatory reaction (Yamashita et al., 2008; Gu et al., 2012). TRAF6 is specifically required for JNK and p38 signaling activation in a Smad-independent way.

However, whether umbilical cord mesenchymal stem cells (UC-MSC) can improve LN by inhibiting TGF-β1 has not been reported. In this study, the therapeutic effects of UC-MSC on LN mice and podocyte protection *in vitro* were investigated, and p-Smad3 and TRAF6 were investigated to explore the potential mechanism of UC-MSC in the treatment of LN.

## 2 Materials and Methods

### 2.1 Establishment of Animal Models

In this study, 3-week-old MRL/Ipr mice (Shanghai Slake Laboratory Animal Co., Ltd.) were used as experimental subjects. MRL/Ipr mice were kept until the 10th week of age. All the animals were kept in an SPF animal room with controlled room humidity (50 ± 10%) and 12-h light/dark cycle with free access to water and food. All animal experiments were performed in accordance with the license by the Yunnan Province Science and Technology Office (Kunming, China), and approval was obtained from the Animal Ethics Committee of Yan’an Hospital Affiliated to Kunming Medical University. All the experiments conformed to the Guidelines for Ethical Conduct in the Care and Use of Animals. We made every effort to minimize stress on animals.

### 2.2 Preparation of Human Umbilical Cord Mesenchymal Stem Cells

**FIGURE 1:**
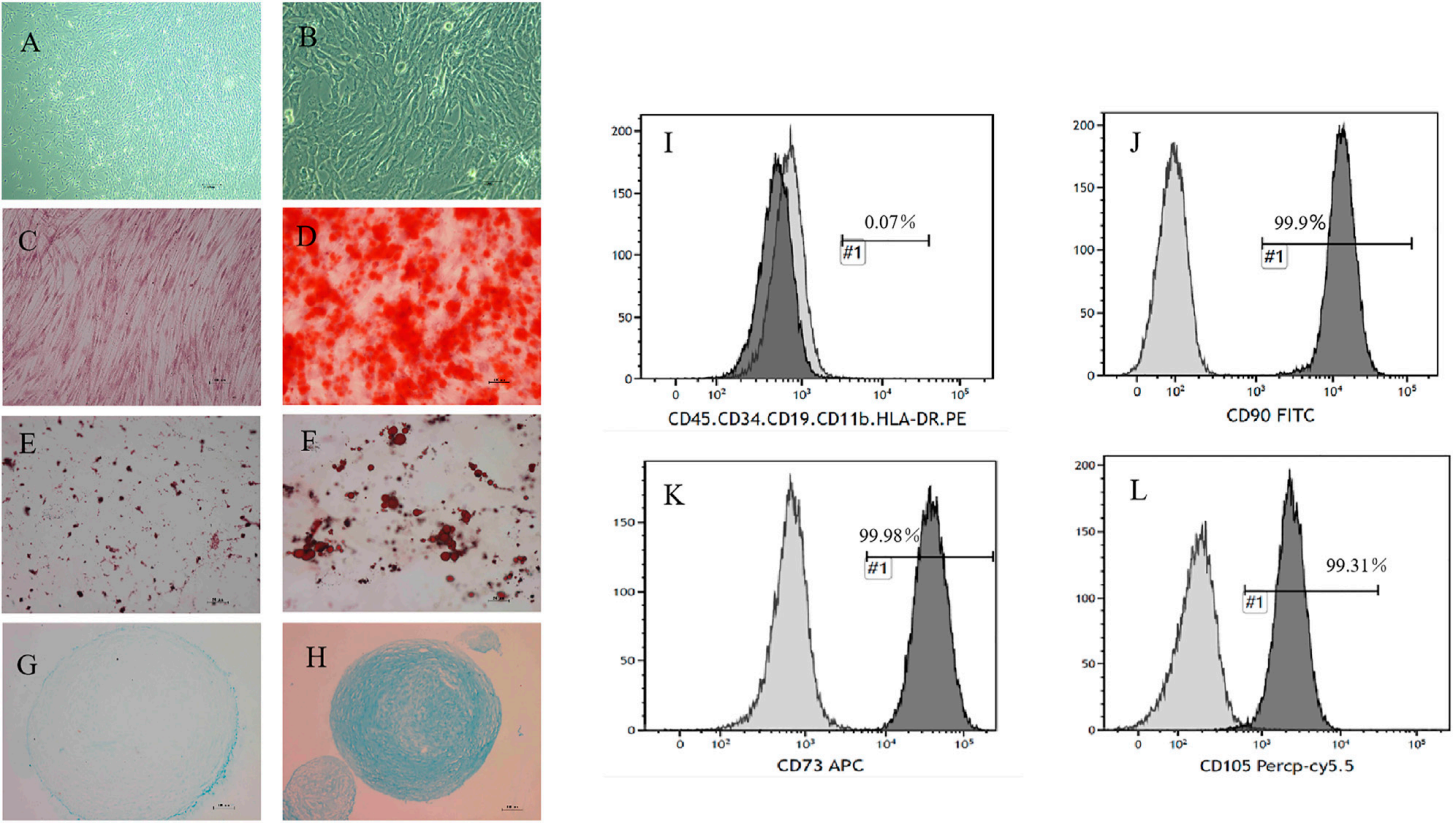
Morphology and phenotype identification of UC-MSC. The morphology of UC-MSC is shown at ×50 magnification **(A)** or at ×200 magnification **(B)**. **(C,D)** UC-MSC were successfully differentiated into osteoblasts, which were verified by Alizarin Red staining. Representative photos were taken of the control group **(C)** and the induction group **(D)** at ×100 magnification. **(E,F)** UC-MSC were successfully differentiated into adipocytes through oil red “O” staining. Representative photos were taken of the control group **(E)** and the induction group **(F)** at ×200 magnification. **(G,H)** UC-MSC were successfully differentiated into the cartilage cells, which were identified by simple blue dye. Representative photos were taken of the control group **(G)** and the induction group **(H)** at ×100 magnification. The surface markers of UC-MSC, including CD90, CD105, CD73, CD45, CD34, CD11b, CD19, and HLA-DR, were analyzed by flow cytometry. Among them, the positive rates of CD90, CD105, and CD73 were above 99%. Others were almost always negatively expressed **(I–L)**.

UC-MSC were prepared and provided by the central laboratory of Yan’an Hospital Affiliated with Kunming Medical University, which is the National Stem Cell Clinical Research Institute in China. Antibodies for labeling the cell surface of UC-MSC, including CD73, CD29, CD45, CD105, CD90, CD79, CD14, and CD34, and the human leukocyte antigen major histocompatibility complex class II molecule DR haplotype (HLA-DR), as well as their isotype controls, were all purchased from BD Bioscience. Cell phenotypes were detected by flow cytometry analysis. In addition, UC-MSC also induced differentiation into osteoblasts, chondrocytes, and adipocytes according to our previous study (Figure 1).

### 2.3 Experimental Group

MRL/Ipr mice were randomly divided into four groups: the LN group, the UC-MSC group, the methylprednisolone (MP) group, and the UC-MSC + MP treatment group. There were six mice in each group, with a total of 30 mice. C57BL/6 mice were used as the normal group at the same age. The treatment is given as follows: (1) UC-MSC group: 2 × 10^5^/100 µL of UC-MSC were administrated by the tail vein at the 10^th^, 12^th^, and 14^th^ weeks. (2) MP group: 30 mg/kg.d of methylprednisolone (MP) was administrated by the tail vein at 14^th^ weeks consecutively for 3 days in a period of treatment (once a day). (3) UC-MSC + MP group: mice were treated as the UC-MSC group, and after the third injection of UC-MSC at 14^th^ weeks, MP injection was subsequently given for three consecutive days at the same dose and method as the MP group (30 mg/kg.d, once a day). (4) Normal group: normal saline were given at equivalent volume. Animals were killed at 16 weeks. Blood was collected, and serum was centrifuged and stored at −80°C. Kidneys were harvested for electron microscope and pathological analyses. The kidney tissue was immediately stored at −80°C for Western blot analyses, embedded in OCT media for immunofluorescence and 10% formalin for the pathological analyses.

### 2.4 Biochemical Measurement

Renal function was evaluated by serum urea nitrogen and creatinine. Renal function was measured by a biochemical instrument (BECKMAN AU5421, Japan). Serum ds-DNA was measured using an ELISA kit (BioVendor).

### 2.5 Renal tissues HE, PAS, Masson stain and electron microscopy analysis

Paraffin embedded renal specimens were cut into 3-μm-thick slices and stained with HE, periodic acid–Schiff (PAS), and Masson’s trichrome and then observed under an Olympus BX-43 microscope.

Small pieces of the renal cortex were prefixed in 3.5% glutaraldehyde, fixed in 1% osmic acid, dehydrated by gradient alcohol and acetone, and embedded in Araldite 618 (Sigma Aldrich, 10,951). Ultrathin sections were counterstained with uranyl acetate and lead citrate and examined with a transmission electron microscope (TEM-1011, Japan).

### 2.6 Immunofluorescence Analysis

The renal tissues were cut into 4-μm-thick frozen sections and incubated overnight at 4°C with a rabbit NPHS2 polyclonal antibody in PBS (1:50). The slices were washed three times with PBS containing 0.01% Triton X-100 and then incubated with anti-rabbit IgG antibodies (1:100) conjugated with Alexa Fluor 594 for 1 h at 37°C. Three 5-min washes were repeated, and the slices were observed under a fluorescence microscope (Nikon DIGITAL SIGHT DS-Ri1).

### 2.7 Western Blot Analysis

Total protein was extracted from the kidney tissue, and the protein concentrations were examined by using a protein assay kit (Bio–Rad, America). Proteins were separated by SDS–PAGE and transferred to PVDF membranes (Millipore, America), blocked, and probed with antibodies against TGF-β1 (1:1,000; Proteintech, America), TRAF6 (1:500; Santa Cruz Biotechnology, America), p-Smad3 (1:1,000; Cell Signaling Technology, America), Smad3 (1:1,000; Cell Signaling Technology, USA), β-actin (1:5,000; Proteintech, America), and Synaptopodin (1:1,000; Proteintech, America). The blots were incubated with secondary antibodies and visualized by super ECL detection reagent (Proteintech, America).

### 2.8 Serum TGF-β1 Determination

The TGF-β1 level in the serum of the normal group, LN model group, and UC-MSC group were detected by using the ELISA kit (NEOBIOSCIENCE).

### 2.9 Cell Culture

An immortalized rat podocyte cell line was purchased from the Beijing Beina Chuanglian Biotechnology Institute (Beijing, China) and cultured in DMEM containing 10% FBS in a humidified atmosphere of 5% CO_2_ in an incubator at 37°C. The cells were seeded in 24-well transwell plates (50,000 cells/well) and exposed to TGF-β1 conditions (8 ng/ml) with or without UC-MSC for 48 h. Cell viability were determined by CCK-8 assays. Additionally, the cells of different groups were harvested to determine synaptopodin protein expression levels by Western blot. All the experiments were repeated three times.

### 2.10 Statistical Analysis

Statistical analyses were performed by SPSS 22.0 software. Measurement data are presented as mean ± SD, and one-way ANOVA were used to compare the differences. *p* < 0.05 was considered statistically significant.

## 3 Results

### 3.1 UC-MSC Therapy Can Improve Renal Function in MRL/Ipr Mice

**FIGURE 2:**
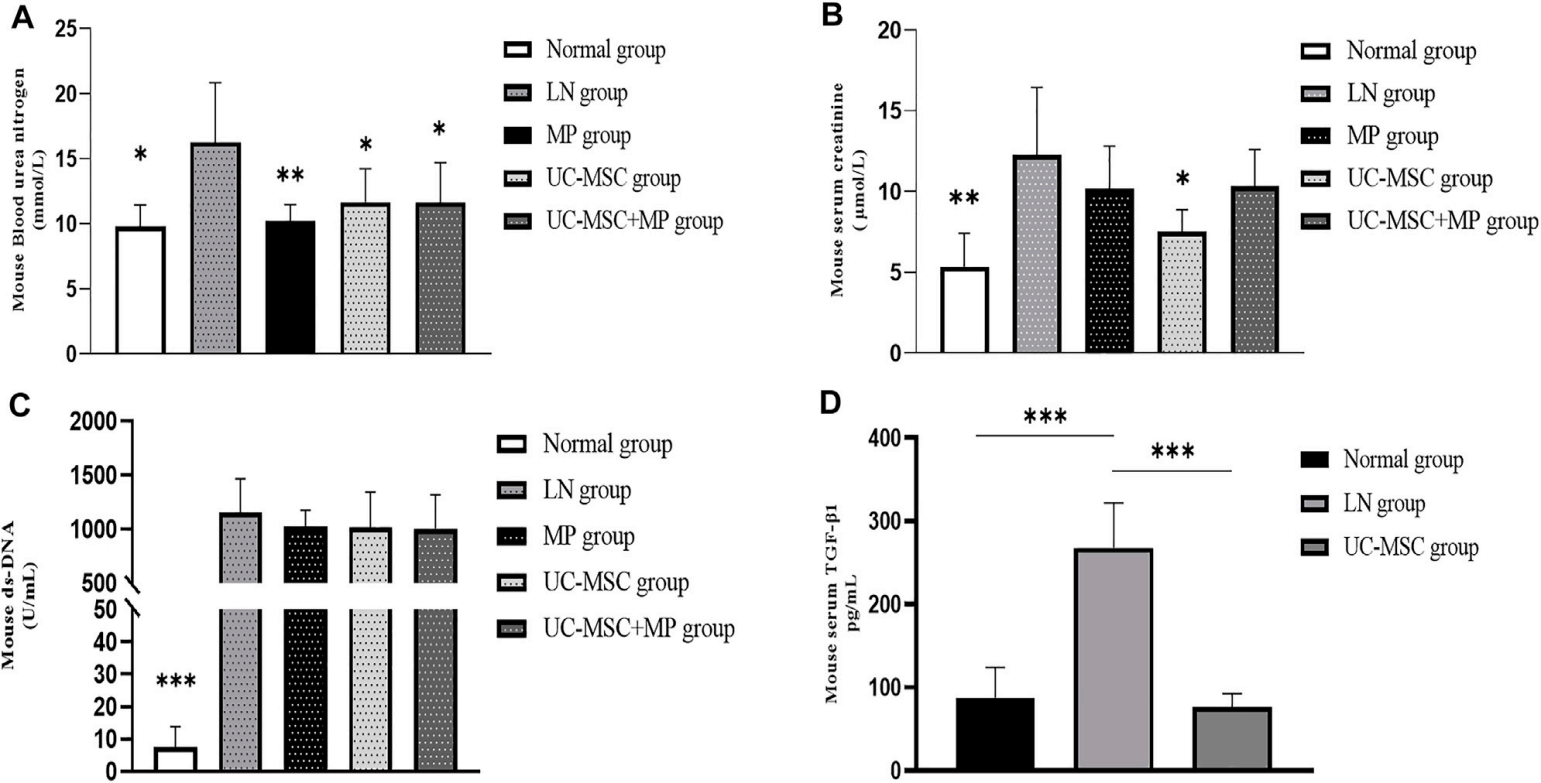
Changes of renal function, serum ds-DNA, and serum TGF-β1 level. Comparison of biochemical measurement. **(A)** Blood serum creatinine level (mmol/L); **(B)** blood urea nitrogen level (μmol/L); **(C)** serum ds-DNA level (U/mL); **(D)** serum TGF-β1 level (pg/ml) vs. LN group: *p < 0.05; **p < 0.01; ***p < 0.001.

Compared with the normal group, the LN group showed significant increases in ds-DNA, creatinine, and urea nitrogen, indicating that the modeling of LN mice was successful. Compared with the LN group, lower urea nitrogen levels were observed in the MP group (*p* < 0.01) and UC-MSC + MP group (*p* < 0.05), and lower levels of creatinine and urea nitrogen (*p* < 0.05) were observed in the UC-MSC group. There were no significant differences between the UC-MSC and MP groups in terms of ds-DNA, creatinine, and urea nitrogen. These results revealed that UC-MSC therapy showed the same effects as MP treatment on improving the renal function in MRL/Ipr mice (Figure 2).

### 3.2 UC-MSC Therapy Improve the Kidney Pathological Injury in MRL/Ipr Mice

**FIGURE 3:**
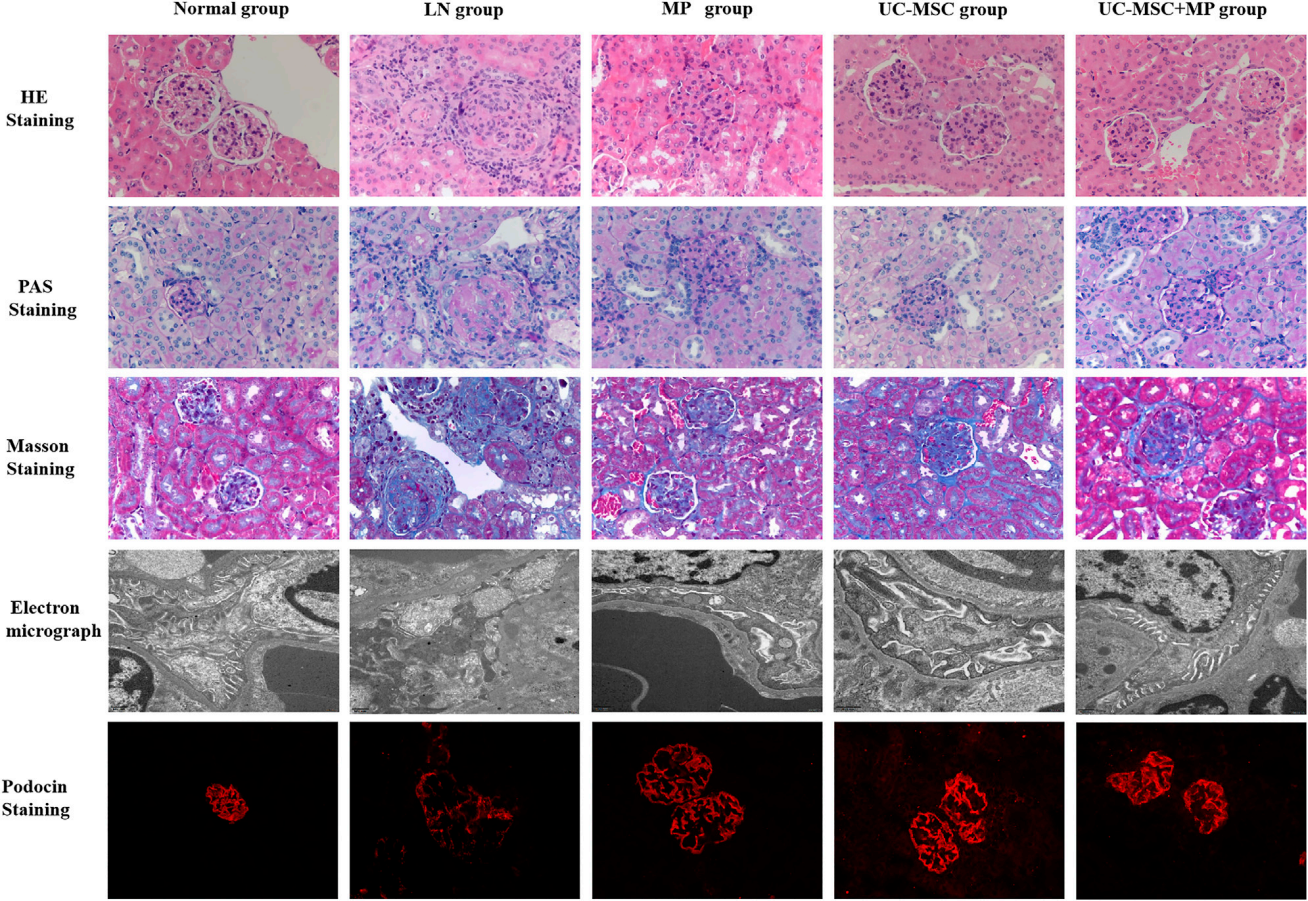
Pathological and electron microscopy results of the renal tissue. HE staining (first line, ×400), PAS staining (second line, ×400), Masson staining (third line, ×400), electron microscopy (fourth line), and podocin immunofluorescence staining (fifth line, ×400). Normal group (first column), LN group (second column), UC-MSC group (third column), MP group (fourth column), and UC-MSC + MP group (fifth column).

Compared with the normal group, the LN group showed an increased glomerular volume, marked glomerular sclerosis, and more inflammatory cell infiltration around the glomeruli in HE staining. PAS staining showed severe proliferation of the mesangial matrix in the glomeruli in the LN group. Masson staining showed obvious glomerular fibrosis in the LN group. Under an electron microscope, obvious foot process fusion, stenosis of the lumen, and swelling of the endothelial cells were observed in the LN group than those in the normal group. A thicker glomerular basement membrane was observed in the LN group (approximately 200–350 nm) than that in the normal group (130–180 nm). The pathological changes of the kidney in the LN group also indicate that the LN model was successful in MRL/Ipr mice. The pathological injury of the kidney in the three treatment groups was improved to some extent as compared with the LN group, which showed that UC-MSC, MP, and their combination could improve the pathological injury of LN mice (Figure 3).

### 3.3 UC-MSC Therapy Restore the Expression of Podocin in Podocytes of MRL/Ipr Mice

Podocin expression was significantly reduced in the LN group as compared with the normal group, indicating the podocyte injury in LN mice. Compared with the LN group, podocin expression was significantly improved in all the treated groups, indicating that UC-MSC, MP, and their combination treatment can improve podocyte injury (Figure 3).

### 3.4 UC-MSC Therapy Improve Renal Injury in MRL/Ipr Mice Through the TGF-β1 Pathway

The serum TGF-β1 level in the LN model group was significantly higher than that in the normal group. A significantly lower serum TGF-β1 level was observed in the UC-MSC treatment group, indicating that UC-MSC might improve kidney injury by inhibiting TGF-β1 (Figure 2).

**FIGURE 4:**
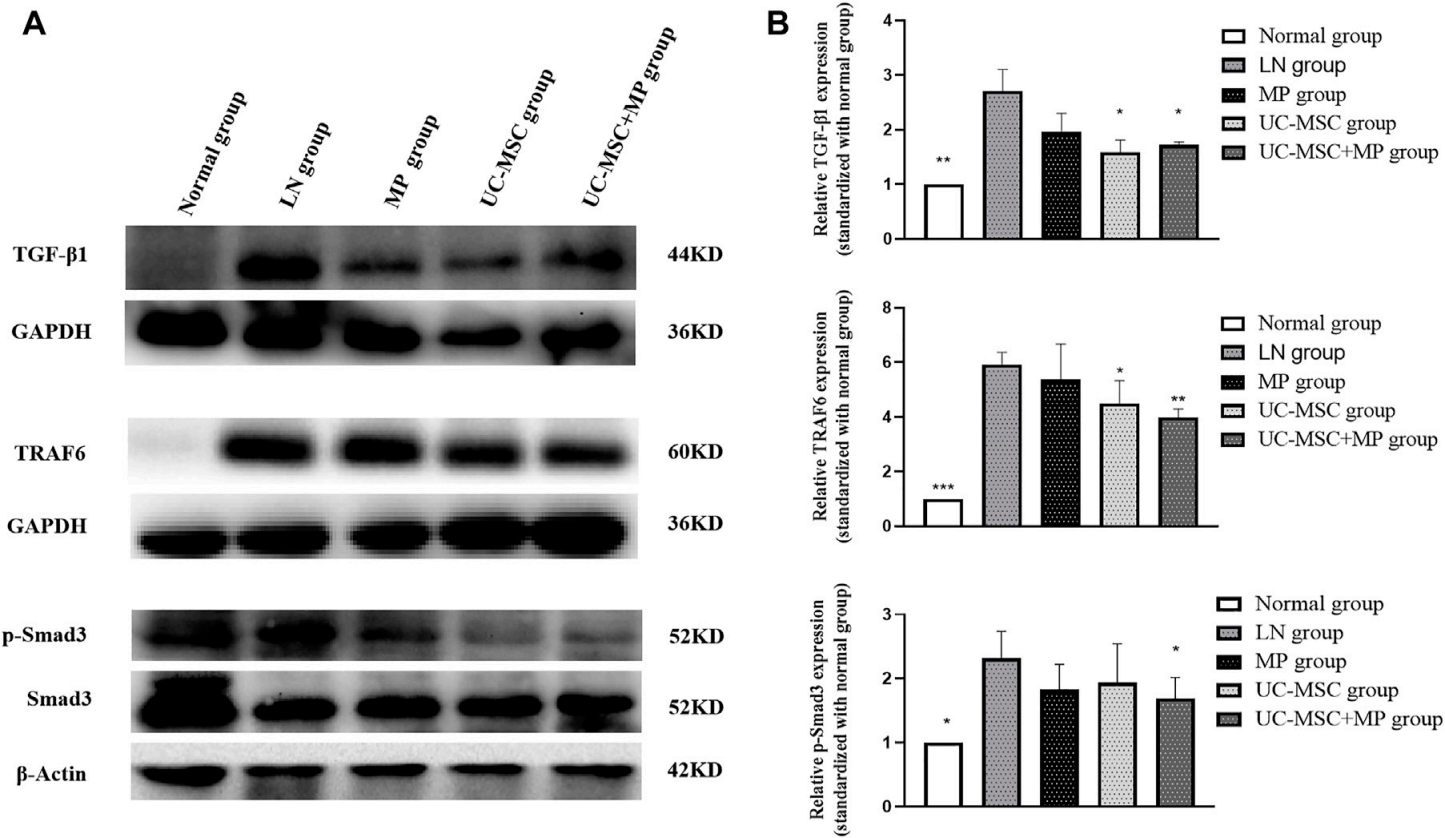
Protein expression of TGF-β1, TRAF6, and p-Smad3 in renal tissues. Western blot results of TGF-β1, TRAF6, and p-Smad3 expression of in renal tissues. **(A)** Western blot results of TGF-β1, TRAF6, and p-Smad3. **(B)** Comparison of TGF-β1, TRAF6, and p-Smad3 protein expression vs. LN group: *p < 0.05; **p < 0.01; ***p < 0.001.

Western blot examination showed significantly increased TGF-β1 expression in the of renal tissues of the LN group than that in the normal group (*p* < 0.05). Interestingly, UC-MSC and UC-MSC + MP groups showed significantly decreased TGF-β1 expression in the kidney than in the LN group (*p* < 0.05). Subsequently, the expression of p-Smad3 and TRAF6, which are the two key proteins of the TGF-β1 downstream pathway, was examined in the renal tissue. The results revealed that p-Smad3 and TRAF6 protein in the LN group were significantly increased compared with the normal group (*p* < 0.05). The downregulation of p-Smad3 and TRAF6 protein was observed after UC-MSC + MP treatment (*p* < 0.05), but only TRAF6 significantly decreased in the UC-MSC group (*p* < 0.05). Those findings suggest that the therapeutic mechanism of UC-MSC in LN mice may involve the inhibition of TGF-β1 (Figure 4).

### 3.5 UC-MSC Therapy Restored Podocyte Activity Inhibited by TGF-β1 *In Vitro*

**FIGURE 5:**
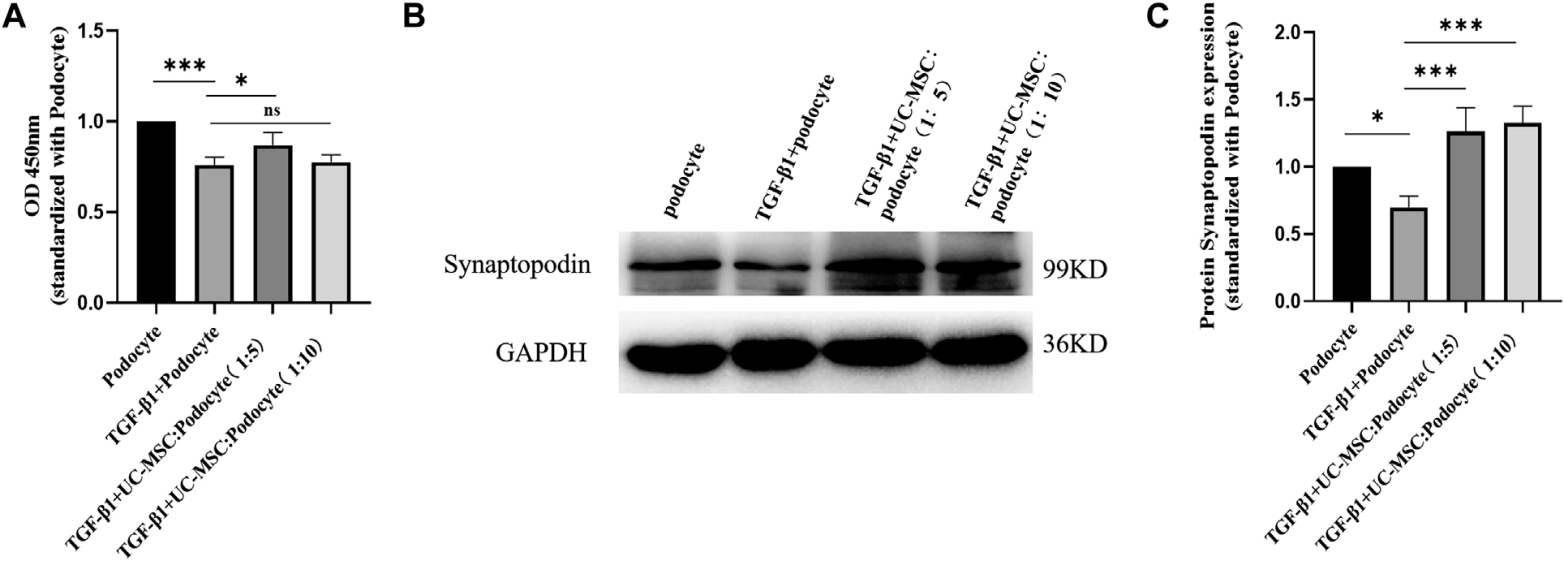
UC-MSC restored activity and synaptopodin expression in podocytes stimulated by TGF-β1. **(A)** Comparison of the podocyte activity. **(B)** Western blot results of synaptopodin. **(C)** Comparison of synaptopodin protein expression. ns: no significance; *p < 0.05; **p < 0.01; ***p < 0.001.

In order to explore the role of TGF-β1 and UC-MSC treatment on podocyte activity *in vitro*, cultured podocyte was stimulated with 8 ng/ml of TGF-β1 with or without UC-MSC, and CCK-8 results showed that podocyte activity was significantly decreased by TGF-β1, while UC-MSC co-culture can restore podocyte activity (*p* < 0.05) (Figure 5).

### 3.6 UC-MSC Restored Synaptopodin Expression in Podocyte Stimulated by TGF-β1 *In Vitro*

Western blot examination was used to explore the role of TGF-β1 and UC-MSC treatment on synaptopodin expression in podocyte *in vitro*. The results revealed that synaptopodin expression significantly decreased in the podocyte subjected to TGF-β1 stimulation (*p* < 0.05), which can be restored by the co-culture with UC-MSC (*p* < 0.05) (Figure 5).

## 4 Discussion

LN is one of the most common manifestations of SLE, and 10% of patients with LN will develop end-stage renal disease (ESRD). Although corticosteroids combined with immunosuppressors are the standard treatment regimens for LN, clinical application of this regimen is still challenging, especially fetal infection. Thus, our study showed that UC-MSC therapy, similar to MP treatment, can improve the renal function and alleviate renal pathological injury in MRL/Ipr mice. The meta-analysis results of MSC treatment in the LN animal model showed lower levels of ANA, ds-DNA, serum creatinine, proteinuria, BUN, and renal sclerosis score in the MSC-treated group (Liu et al., 2018; Zhou et al., 2020), similar to our results. Although the UC-MSC + MP group showed a better effect on the improvement of renal pathological injury than UC-MSC alone group, there was no significant reduction in the serum creatinine level in our study, which may be due to the short follow-up time after MP treatment.

UC-MSC could ameliorate renal injury in LN mice. However, the underlying therapeutic mechanism is unclear. In this study, we found that the expression of podocin, which was one of podocyte-specific markers, was significantly reduced in the renal tissues of LN mice, suggesting the podocyte injury in LN mice. Notably, podocin expression in the renal tissue increased after treatment with UC-MSC, indicating that UC-MSC could improve podocyte injury. Zhang et al. (2019) also reported that UC-MSC promoted the expression of podocin in lupus-prone B6.lpr mice. In addition, their results demonstrated that UC-MSC ameliorated LN by preventing podocyte injury possibly by reducing macrophage infiltration and polarizing macrophages into an anti-inflammatory phenotype. MSC therapy reduced adriamycin-induced podocyte apoptosis, and MSC also restored synaptopodin in mRNA and protein expression in isolated podocytes (Guo et al., 2014). Recent studies revealed the therapeutic effect of MSC on the podocyte injury in diabetic nephropathy not only by their regeneration and differentiation capability but also by secretion of a series of cytokines (Khalilpourfarshbafi et al., 2017). Those studies suggested that MSC have protective effects on podocytes, which is consistent with our results.

Recently, several studies have found that TGF-β1 is involved in the pathogenesis of LN. In this study, the expression levels of TGF-β1 in serum and renal tissues were significantly increased in the LN group but significantly decreased in the UC-MSC treated group, suggesting that UC-MSC treatment may improve LN renal injury through the TGF-β1 pathway. We further explored the downstream targets of TGF-β1 which mainly include the Smad pathway and the Smad-independent pathway. In the context of TGF-β1/Smad signaling, Smad3 is confirmed to be pathogenic because deletion of Smad3 inhibits renal fibrosis. Additionally, TGF-β1 signaling is mediated *via* Smad-independent molecules, including TRAF6, TGF-β-activated kinase 1 (TAK1), p38 mitogen-activated protein kinases (MAPKs), extracellular signal-regulated kinase (ERK), c-JUN N-terminal kinase (JNK), and nuclear factor-κB (NF-κB). TRAF6 is specifically required for JNK and p38 signaling activation in a Smad-independent way. Other researchers found that the upregulation of TRAF6 in the peripheral blood of LN patients increased the possibility of ESRD progression and recurrence within 1 year (Chen et al., 2017). In this study, the expression of p-Smad3 in the renal tissue was significantly increased in the LN model group but significantly inhibited in the UC-MSC + MP group. Meanwhile, the expression of TRAF6, a key protein in the Smad-independent pathway, was inhibited in both the UC-MSC and UC-MSC + MP groups. Our findings suggest that UC-MSC therapy may improve kidney injury in LN by inhibiting the TGF-β1 pathway.

In order to explore the role of TGF-β1 and UC-MSC treatment on the podocyte directly, we conducted *in vitro* experiments, and the results showed that TGF-β1 can affect the activity of the cultured podocyte. Interestingly and notably, UC-MSC intervention can restore the podocyte activity inhibited by TGF-β1 in a transwell co-culture system. Podocytes are terminally differentiated cells that are located on the outer surface of the glomerulus. In addition to maintain the glomerular filtration barrier, they also exhibit immune-like properties (Bhargava and Tsokos, 2019; Frangou et al., 2020). A recent published review by Faul concluded a correlation between the severity of pathological injury in LN patients and intensity of podocyte injury (Faul et al., 2007). Synaptopodin, located on the luminal membrane and alpha-actinin-4 near the basement membrane, maintain the podocyte architecture by the connection with actin (Perysinaki et al., 2011; Rezende et al., 2014; dos Santos et al., 2015; Bollain-Y-Goytia et al., 2011). Levels of synaptopodin, an actin-binding protein involved in stress fiber formation and actin cytoskeleton integrity, decrease during LN (Asanuma et al., 2006). Our study found that the UC-MSC treatment can upregulate the expression of synaptopodin, which also suggested that UC-MSC may improve podocyte injury *in vitro*.

It has been reported that UC-MSC can inhibit TGF-β1 expression in the kidney tissue of the diabetic nephropathy mice model and inhibit its downstream PI3K/Akt and MAPK signaling pathways (Li et al., 2020). Meanwhile, *in vitro* studies revealed that MSC can also attenuate TGF-β1-induced EMT by increasing hepatocyte growth factor (HGF) expression in renal tubular epithelial cells (Wei et al., 2021). MSC have also shown therapeutic effects on renal injury in lupus mice through its interaction with the complement system and immune cells, including Tfh cells, dendritic cells, and B cells (Ma et al., 2013; Ma et al., 2018; Yang et al., 2018).

In our study, UC-MSC therapy was found to alleviate kidney injury, especially podocyte injury in lupus mice, which may be related to the inhibition of the TGF-β1 pathway. The pathogenesis of LN involves diverse mechanisms instigated by elements of the autoimmune response which alter the biology of kidney resident cells. Not only podocytes, mesangial cells, and tubular epithelial cells but also a series of systemic or locally secreted chemokines and cytokines were involved in renal injury during LN and would be affected by MSC. We just focused on podocyte injury in LN, which was the limitation in our study. Further study to explore the mediators of UC-MSC that counteract the TGF-β1-induced podocyte injury or mesangial cells and tubular epithelial cells would be of great value for the UC- MSC therapy.

## Data Availability

The original contributions presented in the study are included in the article/Supplementary Material; further inquiries can be directed to the corresponding authors.
